# The Finely Coordinated Action of SSB and NurA/HerA Complex Strictly Regulates the DNA End Resection Process in *Saccharolobus solfataricus*

**DOI:** 10.3390/ijms23052582

**Published:** 2022-02-26

**Authors:** Mariarosaria De Falco, Alessandra Porritiello, Federica Rota, Viviana Scognamiglio, Amina Antonacci, Giovanni del Monaco, Mariarita De Felice

**Affiliations:** 1 Institute of Biosciences and BioResources, Consiglio Nazionale delle Ricerche, 80131 Naples, Italy; a.porritiello@studenti.unina.it (A.P.); fe.rota@unina.it (F.R.); giovanni.delmonaco@ibbr.cnr.it (G.d.M.); 2 Department of Chemical Sciences and Materials Technologies, Institute of Crystallography, National Research Council, Via Salaria Km 29,300, Monterotondo, 00015 Rome, Italy; viviana.scognamiglio@ic.cnr.it (V.S.); amina.antonacci@ic.cnr.it (A.A.)

**Keywords:** DNA repair, HR, nuclease, DNA helicase, SSB, Archaea

## Abstract

Generation of the 3′ overhang is a critical step during homologous recombination (HR) and replication fork rescue processes. This event is usually performed by a series of DNA nucleases and/or helicases. The nuclease NurA and the ATPase HerA, together with the highly conserved MRE11/RAD50 proteins, play an important role in generating 3′ single-stranded DNA during archaeal HR. Little is known, however, about HerA-NurA function and activation of this fundamental and complicated DNA repair process. Herein, we analyze the functional relationship among NurA, HerA and the single-strand binding protein SSB from *Saccharolubus solfataricus*. We demonstrate that SSB clearly inhibits NurA endonuclease activity and its exonuclease activities also when in combination with HerA. Moreover, we show that SSB binding to DNA is greatly stimulated by the presence of either NurA or NurA/HerA. In addition, if on the one hand NurA binding is not influenced, on the other hand, HerA binding is reduced when SSB is present in the reaction. In accordance with what has been observed, we have shown that HerA helicase activity is not stimulated by SSB. These data suggest that, in archaea, the DNA end resection process is governed by the strictly combined action of NurA, HerA and SSB.

## 1. Introduction

In all organisms, the accurate and faithful duplication of the genomic DNA depends on the joint work of DNA repair and genetic recombination machineries. This interplay is crucial because the replisomes frequently encounter damages that have the potential to stall or collapse a replication fork. DNA double-strand breaks (DSBs) are one of the most cytotoxic and deleterious forms of DNA damage in cells and that can be due to different causes, such as ionizing radiation, reactive oxygen species and chemotherapeutic drugs [1]; however, regardless of their origin, DSBs pose a serious threat and, if not properly repaired, can result in genetic instability leading to the development of cancer or cell death.

The DSBs are repaired mainly by two pathways: non-homologous end joining (NHEJ) and homologous recombination (HR) [2,3]. While NHEJ can occur throughout the cell cycle, HR is limited to S and G2 phases because of the presence of the homologous copy of the damaged DNA. The choice between these two pathways is dictated by a DNA mechanism known as DNA end resection, a tightly regulated machinery that ensures genomic stability [4,5]. During this process, DNA ends are resected through the joint action of helicases and nucleases that unwind the DNA duplex and generate 3′ overhangs required in S and G2 phases for the subsequent repair steps of HR [6,7].

Multiple studies have led to the idea of a two-step, bi-directional model in which the resection is started by the MRN/MRX complex (Mre11, Rad50 and NBS1/XRS2) together with CtIP/Sae2 (functional ortholog of CtIP in budding yeast) that cleaves the 5′ strand DNA away from the DSB ends (Figure S1) [8,9,10,11].

In the second step of resection, the 3′ end is then processed by Mre11 in the 3′-5′ direction [12], whereas the 5′ end is further resected by Exo1 (together with PCNA or the 9-1-1 complex) and Dna2, together with BLM/WRN, RPA and Cdc24 [11,13,14,15,16]. The long 3′-ssDNA tails are coated by multiple copies of the heterotrimeric replication protein A, RPA, (also known as single-strand binding protein, SSB, in bacteria), forming a filament on the ssDNA to prevent the formation of secondary structures and is then replaced by Rad51 that forms a nucleoprotein filament that is necessary for the strand exchange (Figure S1). RPA is also required for promoting the helicase activity of BLM; it binds ssDNA and regulates DNA2 nuclease activity by blocking its 3′-5′ exonuclease activity [17,18]. Thus, EXO1, DNA2, BLM, WRN and RPA constitute the minimal complex that can carry out long-range extensive DNA end resection [19]. All these proteins are evolutionarily highly conserved; indeed, BLM is a RecQ DNA helicase, which is shared by bacteria, eukarya and archaea, while DNA2, which has helicase and nuclease activities, is related to the bacterial RecB proteins.

Several data have demonstrated that in archaea, DNA end resection requires the cooperation between the Mre11–Rad50 and HerA–NurA complexes, which are encoded in the same operon; these proteins work in concert in vitro to process dsDNA [20,21,22,23,24]. HerA and NurA are considered as functional homologs of the eukaryotic Dna2/DNA2, Exo1/EXO1, Sgs1/BLM proteins and therefore the assembled HerA-NurA system serves as a suitable model system for studying HR. However, contradictory results are reported in regard to the enzymatic activities of NurA and HerA from different species, their substrate specificity and their mutual dependence, while all studies clearly showed a physical and functional interaction between the two proteins. For example, whereas NurA from *Pirococcus furiosus* was reported to have no nicking activity [25], *Deinococcus radiodurans* NurA showed HerA-independent nicking endonuclease activity against closed circular DNA molecules [26]. Moreover, while NurA from *Sulfolobus acidocaldarius* was reported to display both ss-endonuclease and 5′-3′ exonuclease activity on ss- and ds-DNA [20], Blackwood et al. [27] were unable to detect nuclease activity by the highly similar NurA from *Saccharolobus solfataricus* in the absence of HerA. Nevertheless, we have demonstrated that NurA from *S. solfataricus* possesses HerA-independent endonuclease and exonuclease activities; however, only the latter is stimulated by HerA. Moreover, we found that NurA nuclease activity is strongly inhibited by ATP, and this might be the reason why our data seem to be in contrast with what was previously observed by Blackwood et al. [28]. In particular, we propose that HerA is able to stimulate the end resection of six nucleotides on a linear DNA substrate suggesting that NurA follows different digestion models in the presence or absence of HerA (as also reported for the *D. radiodurans* protein) [26].

In all organisms, the single-stranded DNA binding proteins (SSBs in bacteria and crenarchaeota, RPAs, Replication Protein A, in eukarya and euryarchaeota) are critical for protecting ssDNA from the action of various enzymes [29,30]. In bacteria, SSB interacts with and stimulates many enzymes involved in DNA metabolism, such as RNA polymerase, exonuclease I, uracil DNA glycosylase, RecQ helicase and priA helicase [31,32,33,34,35]. For instance, SSB was shown to stimulate the 5′-3′ exonucleolytic activity of RecJ on both single- and double-stranded DNA (ssDNA and dsDNA, respectively) substrates in vitro [36,37,38]. Moreover, some in vitro studies show a multifaceted role of *Saccharomyces cerevisiae* RPA in the sequestration of ssDNA generated by DNA unwinding, enhancement of 5′ strand incision and protection of the 3′ strand [7]. In addition, interestingly, RPA is also required both to direct Dna2 nucleolytic activity to the 5′-terminated strand of the DNA break and to inhibit 3′-5′ degradation by Dna2, actions that generate and protect the 3′-ssDNA overhangs, respectively [11]. Interestingly, Wei et al. demonstrated that *S. tokodaii* StoNurA physically interacts with StoSSB, and its exo- and endo-nuclease activities are inhibited by it [39]. All these data suggest that RPA/SSB has an important role in DSBs resections through modulating the nuclease-helicase complex.

We have previously demonstrated that NurA is endowed with exo- and endonuclease activities that are not strictly dependent on the presence of HerA; in particular, the endo- and exonuclease activities have distinct requirements: whereas the exonuclease activity is stimulated by HerA and depends on the catalytic D58 residue, the endonuclease activity is HerA-independent and is not affected by the D58A mutation [28]. In addition, NurA/HerA exonuclease activity is inhibited by the *S. solfataricus* RecQ-like Hel112 helicase. In contrast, the endonuclease activity of NurA is not affected by the presence of Hel112 [40].

In order to gain further insight into *S. solfataricus* NurA and NurA/HerA complex roles in the DNA end resection process, we performed an in vitro characterization of their DNA nuclease, helicase and DNA binding activities in combination with *S. solfataricus* SSB protein. The results obtained indicate that SSB inhibits NurA endo- and exonuclease activities on various substrates; in particular, the inhibition of exonuclease activity is greater the shorter the length of the oligonucleotide. Moreover, NurA/HerA stimulates SSB binding onto DNA, whereas the latter does not influence NurA-DNA binding. Interestingly, HerA reduces its binding to the DNA in the presence of SSB, but it is not influenced by the latter when the oligonucleotide used in the reaction has a fork-like structure. Nevertheless, the helicase activity of HerA, which is barely detectable even in combination with NurA, does not show any significant increase when SSB is added to the reaction.

Based on our results, we propose a hypothetical model of DNA end resection mechanism in *S. solfataricus*, which could lead to a better understanding of this process even in higher organisms, such as eukaryotes.

## 2. Results and Discussion

### 2.1. SSB Effect on NurA Endonuclease Activity

**Figure 1:**
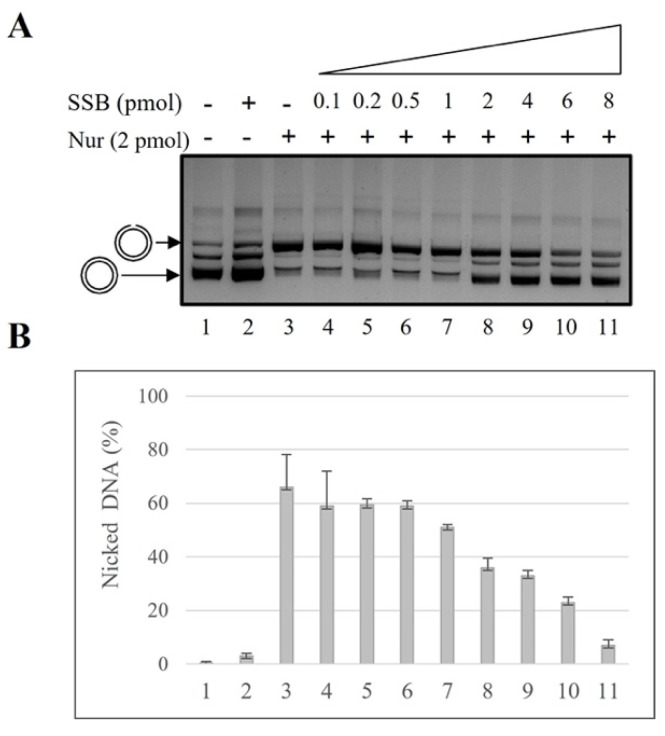
NurA nicking activity is inhibited by SSB. (**A**) NurA nicking activity has been tested on a pBlueScript plasmid as circular double-stranded DNA substrate. Negative controls were performed in absence of proteins (lane 1) and in the presence of 8 pmol of SSB alone (lane 2). NurA endonuclease activity was analyzed in the absence (lane 3) and in the presence of increasing amounts of SSB (lanes 4–11). (**B**) The gels were quantified as reported in Materials and Methods, and the values obtained (average of three different experiments) are reported here.

It has been suggested that NurA may be the functional homolog of the eukaryotic Dna2/DNA2 and Exo1/EXO1; those two enzymes are indispensable to ensure a long end resection (up to 100 nt) during the homologous recombination process [41]. The long 3′-ssDNA tails were generated then coated by multiple copies of the replication protein A (RPA) that form a filament on the ssDNA to prevent the formation of secondary structures. Our previous results revealed that, in vitro, *S. solfataricus* NurA possesses exonuclease and single-strand nicking activity that relies on two different active sites of the protein. Moreover, we demonstrated that while the exonuclease activity is considerably increased by HerA, the nicking activity is not [28]. Considering the significant involvement of RPA in homologous recombination, we decided to determine whether the *S. solfataricus* SSB may influence NurA endonuclease activity. As previously demonstrated [28], HerA neither stimulates nor inhibits NurA endonuclease activity, so we tested the role of SSB on NurA activity on a circular double-stranded DNA. As shown in Figure 1, we analyzed NurA nicking activity using a circular double-strand DNA molecule of about 3000 bp (see Materials and Methods) using increasing amounts of SSB and a fixed concentration of NurA (Figure 1).

NurA, as expected, is endowed with single-strand nuclease activity on double-strand DNA (nicking); indeed, in these conditions, 2 pmoles of the protein were able to convert about 66% of the substrate into a nicked product (Figure 1A, lane 3 and Figure 1B, bar 3); nevertheless, the presence of increasing amounts of SSB significantly inhibits NurA endonuclease activity (lanes 4–11). In particular, the SSB effect on NurA was detectable starting from 2 pmoles of SSB and reached a maximum value at 8 pmoles where the residual nicking activity result was about 7%, indicating that SSB inhibition on NurA endonuclease activity starts when they are in a stoichiometric ratio of 1:1 (about 2 pmoles of each protein and about 250 pmoles of dsDNA, lane 8). Considering the fact that the substrate is a double-stranded DNA and is in a much higher molar ratio than SSB, we can exclude that the inhibition effect is due to the seizing of SSB; rather, it is a direct effect of SSB on the endonuclease activity of NurA.

We also tested the effect of SSB on the nicking activity of NurA/HerA; the results obtained indicate that the nicking activity is inhibited, even in the presence of HerA (Figure S2).

### 2.2. SSB Effect on NurA Exonuclease Activity on Different DNA Substrates

**Figure 2:**
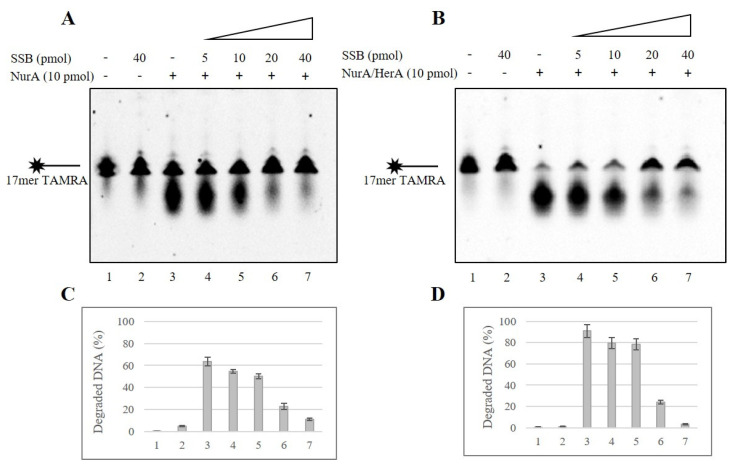
Effect of SSB on NurA and NurA/HerA exonuclease activity on 17 mer ssDNA substrate. (**A**) NurA exonuclease activity was tested on a 17 mer 5′-TAMRA ssDNA in absence (lane 3) or presence (lanes 4–7) of increasing amounts of SSB. Negative controls were performed in absence of proteins (lane 1) and in the presence of the maximum amount (40 pmol) of SSB alone (lane 2). (**B**) The same experiment was performed using 10 pmoles of NurA/HerA complex instead of NurA. (**C**,**D**) The gels were quantified as reported in the Materials and Methods, and the values were average of three different experiments and reported here.

In order to get a deeper insight into how SSB may influence NurA and NurA/HerA functions, we first analyzed the exonuclease activity of NurA in the absence of HerA. A 17 mer oligonucleotide was used as the substrate to analyze the effect of SSB on NurA exonuclease activity. As indicated in Figure 2, NurA exonuclease activity was reduced, starting from 5 pmoles of SSB, and reached 19% inhibition when 40 pmoles of SSB were present in the assay (Figure 2A–D, lanes 4–7). Considering that the DNA is used in a much higher molar ratio in respect of the proteins, we can exclude that the observed effect is due to the seizing of the DNA molecules by SSB. In addition, as shown in Figure 2, lanes 6 and 7 (A–D), the maximum inhibition was obtained when the two proteins were in a molar ratio between 1:2 and 1:4.

Therefore, we analyzed the effect of SSB on the NurA/HerA complex using the same substrate. As shown, the NurA/HerA complex displayed an exonuclease activity of about 91%; when SSB was added to the reaction, a conspicuous inhibition was observed—SSB decreased its activity up to 97%, causing a reduction of the nuclease activity from 91% to 3% (Figure 2B,D, lanes 3 and 7).

**Figure 3:**
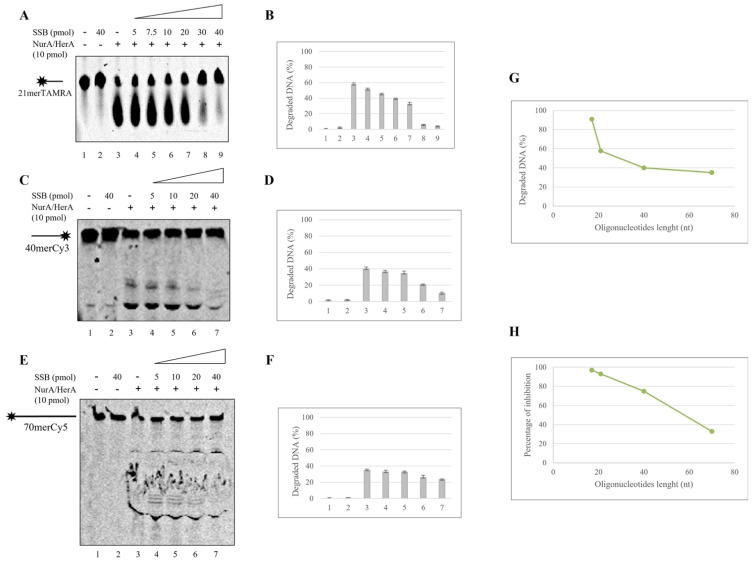
Effect of SSB on NurA/HerA exonuclease activity on various DNA substrates different in length. NurA/HerA exonuclease activity was tested on a 21 merTAMRA (**A**), on a 40 merCy3 (**C**) and on a 70 merCy5 (**E**) oligonucleotides in absence (lanes 3) or presence (lanes 4–9 for 21 merTAMRA; lanes 4–7 for 40 merCy3 and 70 merCy5) of increasing amounts of SSB oligonucleotide. Negative controls were performed in absence of proteins (lane 1) and presence of 40 pmol of SSB alone (lane 2). (**B**,**D**,**F**) Values obtained from the quantization of the activity on each different substrate, 21 merTAMRA (**B**), 40 merCy3 (**D**) and 70 merCy5 (**F**), are reported here; the values are the average of three different experiments. (**G**) The graph shows the trend of NurA/HerA nuclease activity (%) related to the substrate length used. (**H**) The graph shows the trend of SSB inhibition on NurA/HerA nuclease activity related to the length of the substrate analyzed.

As shown in Figure 3, we made use of various single-stranded oligonucleotides differing in length: 21 mer, 40 mer and 70 mer; for the oligonucleotides sequences, see Section 3, Table 1. SSB inhibits the exonuclease activity of NurA/HerA complex on each substrate analyzed, indeed it went from 58% to 4% on 21 merTAMRA (Figure 3A,B), from 40% to 10% on 40 merCy3 (Figure 3C,D) and, on 70 merCy5, the longest substrate used, it went from 35% to 23% (Figure 3E,F). A striking observation was that the exonuclease activity of the complex decreased with the increase of substrate length; indeed, it went from 91% for the 17 mer to 35% for the 70 mer (Figure 3G). In addition, the inhibition of SSB on the exonuclease activity of the NurA/HerA complex decreased as the length of the substrate increased, going from 97% for 17 mer to 33% for 70 mer (Figure 3H).

### 2.3. Mutual Influence of NurA, HerA and SSB on Their Binding onto the DNA

To better understand the mechanism underlying the observed inhibition of SSB on NurA and NurA/HerA nuclease activity, we decided to analyze the reciprocal effect of the three proteins on their binding onto DNA.

**Figure 4:**
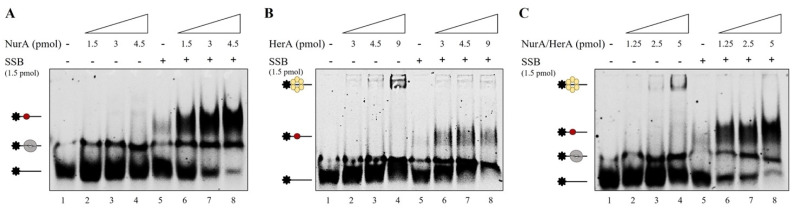
NurA, HerA and SSB reciprocal effect on their binding onto DNA. NurA (grey donut); HerA (yellow hexamer); SSB (red dot). (**A**) Increasing amounts of NurA were analyzed for their DNA binding affinity for a 17 merTAMRA oligonucleotide (lanes 2–4). A fixed amount of SSB was incubated with the oligonucleotide in absence (lane 5) and in presence of the aforementioned quantity of NurA (lanes 6–8). (**B**) The same experiment was performed to analyze HerA and SSB reciprocal effect on the binding onto the 17 merTAMRA oligonucleotide. Three different amounts of HerA were tested for their binding onto DNA (lane 2–4); afterward, a single amount of SSB was analyzed (lane 5), and the latter was mixed with the aforementioned quantity of HerA (lanes 6–8). (**C**) NurA/HerA complex and SSB joint effect on their binding onto DNA. Increasing amounts of NurA/HerA complex were analyzed on their binding to 17 merTAMRA oligonucleotide in the absence (lanes 2–4) and presence (lanes 6–8) of a fixed quantity of SSB (1.5 pmoles). Controls were run in lane 1, where the oligonucleotide was incubated with no proteins and lane 5, where 1.5 pmoles of SSB alone were analyzed for their binding onto DNA.

To this aim, we performed EMSA assays using diverse oligonucleotides, different in length and/or structure (see Section 3 Table 1). The proteins and the DNA were added to the reaction in all possible orders of combination, but the results always showed the same effect, even when NurA or NurA/HerA were preincubated with the DNA (data not shown). Therefore, we decided to incubate the proteins for 5 min at room temperature in order to allow their interaction; only then did we add the indicated oligonucleotides and prolong the room temperature incubation for another 10 min. First, we analyzed the binding of increasing amounts of NurA onto a 17 merTAMRA (Figure 4, lanes 2–4), then we used a fixed concentration of SSB (Figure 4A, lane 5) and finally, the combination of SSB and the three different amounts of NurA (Figure 4, lanes 6–8).

Surprisingly, NurA drastically stimulated SSB binding onto DNA; the resulting shift is indicated by a black line bound to a red dot (Figure 4A, compare lanes 5 with 6–8). The same analysis was performed with HerA (Figure 4B), and still, a stimulation of SSB binding onto DNA was observed, but the effect of HerA was very small compared to that of NurA (Figure 4B, compare lanes 5 with 6–8). Interestingly, a reduction of HerA shift, indicated by the black line with the yellow hexamer, was observed when SSB was present in the reaction (Figure 4B, compare lanes 2–4 with 6–8). These data suggest that the binding of SSB onto the DNA, on one hand, is stimulated by NurA, but at the same time reduced the binding affinity of HerA for the single-stranded DNA, probably allowing it to bind a DNA structure in order to unwind it. Finally, we analyzed the behavior of SSB in combination with NurA/HerA complex (Figure 4C); also, in this case, SSB binding onto DNA is remarkably stimulated by the presence of the complex and, again, a breakdown of the HerA bound to the DNA was observed. The binding of NurA (black line with grey donut) seems not to be significantly influenced by the presence of the other proteins.

**Figure 5:**
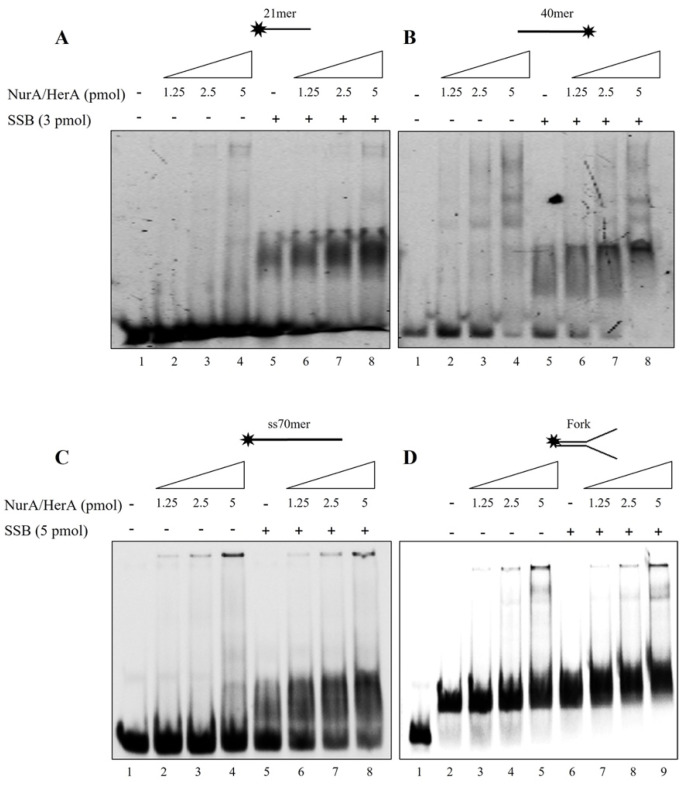
NurA/HerA and SSB mutual effect on different DNA substrates. (**A**) Increasing amounts of NurA/HerA complex were analyzed on a 21mer oligonucleotide in absence (lanes 2–4) and presence (lanes 6–8) of a fixed concentration of SSB (3 or 5 pmoles). Lane 5 refers to SSB binding to DNA, while lane 1 represents a negative control where no proteins were added in the reaction. The same assay conditions were performed using 40mer (**B**), 70mer (**C**) oligonucleotides and a forked DNA structure (**D**).

We, therefore, decided to analyze the behavior of NurA/HerA and SSB using substrates different in length and/or structure (Figure 5).

**Figure 6:**
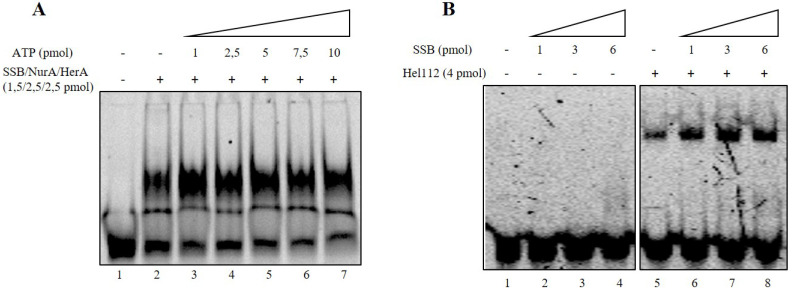
(**A**) Effect of ATP on NurA/HerA/SSB binding onto DNA. A fixed amount of NurA, HerA and SSB were analyzed for their binding onto DNA in absence (lane 2) and presence of increasing amounts of ATP (lanes 3–7). Lane 1 represents a negative control where no proteins were added to the reaction. (**B**) Hel112 effect on SSB binding onto DNA. Increasing amounts of SSB were analyzed for their binding onto DNA in the absence (lanes 2–4) or presence (lanes 6–8) of Hel112. Lane 5 refers to Hel112 binding to DNA, while lane 1 represents a negative control where no proteins were added to the reaction.

In all the different cases analyzed, SSB binding was strongly stimulated by the presence of NurA/HerA; in addition, surprisingly, HerA did not reduce its binding to DNA when a fork structure was used in the reaction, contrary to what was observed for all other substrates analyzed. These results strengthen our hypothesis, according to which HerA, in the presence of SSB, was forced to leave the single-stranded DNA and bind to the forked structures in order to start, eventually, its unwinding activity. We also analyzed the effect of ATP on NurA/HerA influence on SSB by performing the band shift assay on a 17 mer oligonucleotide using 2.5 pmoles of NurA/HerA complex and 1.5 pmoles of SSB in the absence or presence of increasing amounts of ATP. The results indicate that ATP had no effect on the stimulation of SSB binding to DNA by NurA/HerA (Figure 6A). To confirm that the stimulation was a specific effect of NurA/HerA on SSB, we performed band shift assays under the same experimental conditions using 4 pmoles of Hel112 and increasing amounts of SSB. Hel112 is a RecQ-like DNA helicase from *S. solfataricus*, which is endowed with both DNA unwinding and annealing activities [42,43]. We demonstrated that Hel112 does not influence SSB binding to DNA, unequivocally indicating that the stimulation of SSB binding is strictly due to the action of the NurA/HerA complex (Figure 6B).

### 2.4. SSB Does Not Have Any Effect on HerA Helicase Activity

**Figure 7:**
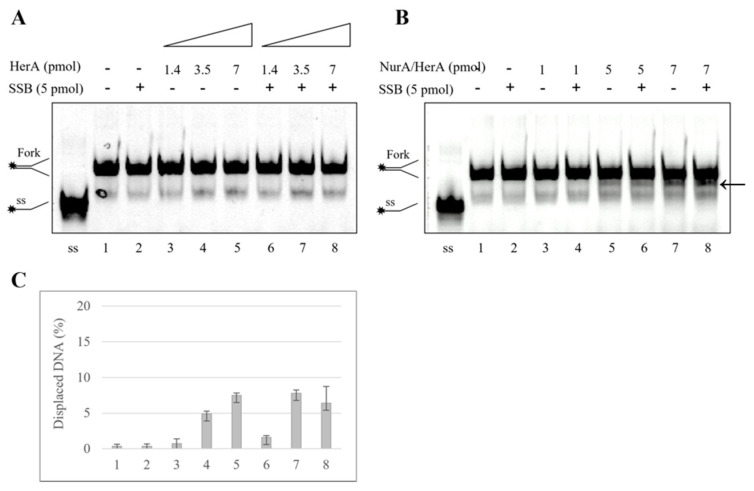
Helicase activity of HerA or NurA/HerA complex on a 70 merCy5 forked substrate. (**A**) Increasing amounts of HerA (from 1,4 to 7 pmoles) were analyzed for helicase activity in absence (lanes 3–5) and presence (lanes 6–8) of 5 pmoles of SSB. (**B**) Increasing amounts of NurA/HerA complex (from 1 to 7 pmoles) were analyzed for helicase activity in absence (lanes 3, 5 and 7) and presence (lanes 4, 6 and 8) of 5 pmoles of SSB. Lanes 1 and 2 are negative controls in which no proteins and only SSB were used in the reaction, respectively. ss refers to 70 merCy5 single-strand oligonucleotide migration. The arrow indicates the degradation products due to NurA nuclease activity. (**C**) The gels have been quantified as reported in Materials and Methods, and the average of three different experiments is reported.

Several biochemical characterizations revealed that HerA is a helicase that exhibits ATPase activity [20,21,23,44] and that this activity is strictly dependent on the presence of NurA [27]. Nevertheless, in our experimental conditions, we never observed clear helicase activity by *S. solfataricus* HerA—neither in the presence nor absence of NurA [28]. Since Lee et al. [45] demonstrated that WRN helicase activity is differently modulated by RPA in a concentration-dependent manner, we decided to analyze the possible effect of SSB on HerA. As shown in Figure 7A, increasing amounts of HerA showed a barely detectable DNA helicase activity (Figure 7A,C, lanes 3–5) that is not significantly stimulated by the presence of SSB (Figure 7A,C, lanes 6–8). We then investigated the effect of SSB on NurA/HerA helicase activity (Figure 7B). We analyzed three different concentrations of NurA/HerA complex (1, 5 and 10 pmoles) in the absence (lanes 3, 5 and 7) or presence of SSB (lanes 4, 6 and 8). Furthermore, in this case, we were not able to detect any HerA helicase activity, even in complex with NurA and in the presence of SSB. In order to exclude that the lack of HerA helicase activity was due to a possible mis-folding of the proteins, we performed *Circular Dichroism* analysis (see Supplementary Materials). The results obtained confirmed that the three proteins were in a correct three-dimensional conformation, indicating that the absence of DNA helicase activity cannot be attributed to a mis-folding of HerA and its interacting proteins. Furthermore, in Figure 7B, it is possible to observe a weak nuclease activity, pointed out by the arrow, due to the presence of NurA. As already demonstrated by our group [28], the nuclease activity of NurA requires Mn^2+^ ions even if Mg^2+^ ions, which are present in the helicase buffer (see Section 3), enable a faint nuclease activity.

### 2.5. SSB Showed No Interaction with NurA

**Figure 8:**
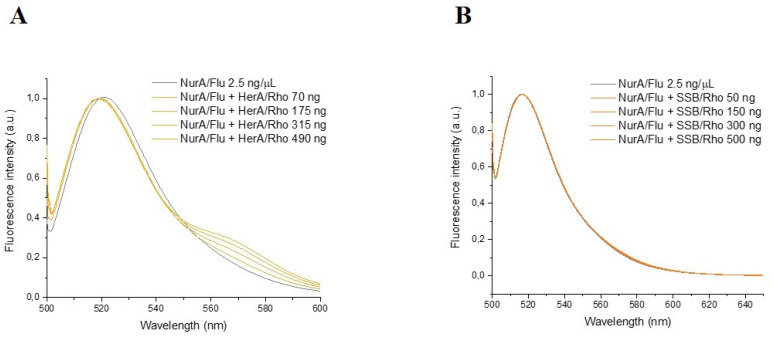
(**A**) Emission spectra of NurA labeled with fluorescein 5(6)-isothiocyanate in the presence of increasing concentrations of HerA labeled with rhodamine B. (**B**) Emission spectra of NurA labeled with fluorescein 5(6)-isothiocyanate in the presence of increasing concentrations of SSB labeled with rhodamine B. Buffer: 1 mM sodium phosphate buffer pH 7.0.

In order to perform FRET measurements, fluorescein 5(6)-isothiocyanate and rhodamine B were selected as donor-acceptor pair fluorophores because fluorescence emissions of the donor overlap with the absorption/excitation spectrum of the acceptor. Figure 8 reports the emission spectra of NurA labeled with fluorescein 5(6)-isothiocyanate in the presence of HerA labeled with rhodamine B as well as SSB labeled with rhodamine B. As shown, there is an increase in fluorescence emission of ca. 50% at 570 nm upon addition of HerA labeled with rhodamine B (Figure 8A), highlighting that a fluorescence energy transfer between the probes occurred, thus confirming an interaction between the two proteins. Surprisingly, despite the functional interaction observed in the experiments described above, any increase of the fluorescence emission of rhodamine appeared at 570 nm when NurA labeled with fluorescein 5(6)-isothiocyanate was incubated with SSB labeled with rhodamine B (Figure 8B), suggesting that, in these experimental conditions, there appeared to be no physical interaction between NurA and SSB.

## 3. Materials and Methods

### 3.1. Chemicals

Fluorescein 5(6)-isothiocyanate, rhodamine B, sodium phosphate, DMSO were purchased from Merck.

### 3.2. Expression and Purification of Recombinant Proteins

All chromatographic separations were performed on ÄKTA FPLC systems (GE Healthcare, Buckinghamshire, UK); protein concentration was determined with a Bio-Rad protein assay kit (Bio-Rad, Hercules, CA, USA), and purity was assessed by SDS-PAGE. Before purification, all cell extracts were extensively digested with DNaseI, and the absence of contaminant nucleic acids in purified protein preparations was always assessed by ethidium bromide-stained agarose gel electrophoresis.

The gene coding for recombinant NurA and HerA were obtained, as previously described [28]. The *E. coli* BL21-CodonPlus(DE3)-RIL cells (Novagen, Darmstadt, Germany), transformed with the plasmid of interest, were grown at 37 °C in 500 mL of LB (Luria–Bertani) medium containing 30 μg/mL chloramphenicol and 30 μg/mL kanamycin. When the culture reached an A_600_ of 0.6 OD, protein expression was induced by the addition of IPTG 0.2 mM. The bacterial culture was then incubated for 3 h at 37 °C. The cells were harvested by centrifugation, and the pellet was resuspended in 10 mL of Buffer A (25 mM Tris-HCl pH 8, 2.5 mM MgCl_2_, 100 mM NaCl), supplemented with protease inhibitors (Complete Mini EDTA-free, Roche, Basil, Switzerland). The cells (1 g) were broken by three consecutive passages through a French pressure cell apparatus (Aminco Co., Silver Spring, MD, USA) at 1500 p.s.i. The resulting lysate was centrifuged for 30 min at 20,000 r.p.m. (Beckman, Pasadena, CA, USA; rotor JA-25.50) at 4 °C. The supernatant was heat-treated at 70 °C for 20 min and centrifuged for 30 min at 20,000 r.p.m. (Beckman rotor JA-25.50) at 4 °C. The soluble fraction was subsequently filtered through a 0.22 μm filter (Merck Millipore, Burlington, MA, USA) and loaded onto a Mono Q HR 10/100 column (GE Healthcare Life Sciences, Marlborough, MA, USA). Fractions of 0.5 mL were collected and analyzed by SDS/PAGE, and those containing the recombinant protein were pooled and concentrated up to 0.8 mg/mL for NurA and 1 mg/mL for HerA, using an Amicon system.

The *E. coli* BL21-CodonPlus(DE3)-RIL cells (Novagen) transformed with the pET19b-SSB [46], were grown at 37 °C in 500 mL of LB (Luria–Bertani) medium containing 30 μg/mL chloramphenicol and 30 μg/mL kanamycin. When the culture reached an A_600_ of 0.6 OD, protein expression was induced by adding isopropyl β-d-thiogalactoside at a concentration of 1 mM. The bacterial culture was incubated at 37 °C for an additional 3 h. Cells were then harvested by centrifugation and the pellet (1 g) resuspended in 10 mL of buffer A (50 mM Tris/HCl pH 8, 50 mM NaCl, 10% glycerol) supplemented with a protease inhibitor cocktail (Complete Mini EDTA-free Roche). Cells were broken by two consecutive passages through a French pressure cell apparatus (Aminco Co) at 1500 p.s.i. The sample was centrifuged for 30 min at 20,000 r.p.m. (Beckman rotor JA-25.50) at 4 °C. The supernatant was subjected to heat-treatment at 70 °C for 20 min. The thermo-precipitated proteins were removed by centrifugation for 30 min at 20,000 r.p.m. (Beckman rotor JA-25.50) at 4 °C. The supernatant was passed through a 0.22 μm filter (Millipore) and loaded onto a Hi-Trap Heparin column (1 mL column volume) at 0.5 mL/min. The protein was eluted using a linear gradient of NaCl (from 0.1 to 1 M NaCl) at 0.5 mL/min for 30 min. Fractions containing the recombinant protein were analyzed by SDS/PAGE, pooled and concentrated up to 1 mg/mL using an Amicon system.

### 3.3. Substrate Preparation

The Forked substrate was generated annealing a 70 lagFork oligonucleotide with 70 leadCy5 (see Table 1). The circular plasmid used to analyze the nicking activity of NurA was pBlueScript.

**Table 1:** Oligonucleotides used for the assays.

Name	Sequence
17 merTAMRA	5′-GTTTTCCCAGTCACGAC-3′
21 merTAMRA	5′-GCTATCGTACATGATATCCTC-3′
40 merCy3	5′-GCCGTGATCACCAATGCAGATTGACGAACCTTTGCCCACGT-3′
70 lagFork	5′-TTTTTTTTTTTTTTTTTTTTCACACTCACTTAAGCCGAATTCTTAGGGTTAGGGTTAACATCAAGTCACG-3′
70 leadCy5	5′-CGTGACTTGATGTTAACCCTAACCCTAAGAATTCGGCTTAAGTGAGTGTGAGGATATCATGTACGATAGC-3′

### 3.4. Endonuclease Assay

Endonuclease activity of NurA was assayed on a 3000 bp circular DNA (PBlueScript). Reactions were performed in 10 µL reaction volume, containing 200 mM HEPES/NaOH pH 7.5, 10 mM β-mercaptoethanol, 300 mM NaCl, 50 mM MnCl_2_ and incubated for 30 min at 70 °C in a heated-top PCR machine to prevent evaporation. The reactions were stopped by the addition of 0.5% SDS, 40 mM EDTA, 0.5 mg/mL proteinase K, 20% glycerol and the products were separated on a 1% agarose 0.5 × TBE, stained with ethidium bromide and visualized under UV light by VersaDocTM MP 1000 system and quantified using ImageJ 1.5 as the processing program. The enzymatic product values were calculated considering the intensity of the product versus the intensity of the remaining substrate.

### 3.5. Exonuclease Assay

The exonuclease activity of NurA and HerA/NurA complex was assayed on a variety of DNA substrates reported in Table 1. Reactions were performed in 10 µL reaction volume, containing 200 mM HEPES/NaOH pH 7.5, 10 mM β-mercaptoethanol, 300 mM NaCl, 50 mM MnCl_2_ and incubated for 30 min at 70 °C in a heated-top PCR machine to prevent evaporation. Reactions were terminated by the addition of 5 μL of stop solution (10 mM EDTA, 98% formamide) and separated on 15% polyacrylamide gel (19:1) and 8 M urea in 1 x TBE. Gels were visualized by VersaDoc^TM^ MP 4000 system (Bio-Rad) and quantified using ImageJ 1.5 as processing program. The enzymatic products values were calculated considering the intensity of the product versus the intensity of the remaining substrate.

### 3.6. DNA Band-Shift Assays

For each substrate, reactions were performed in a final volume of 10 μL containing 5 pmoles of fluorescent DNA in 20 mM HEPES/NaOH pH 7.5, 35 mM β-mercaptoethanol, 500 mM NaCl, 50 mM MgCl_2_ and the indicated amounts of the different proteins. Following incubation for 10 min at room temperature, 1 μL of 100% glycerol was added and the complexes were separated by electrophoresis through 5% polyacrylamide (29:1) gels in 0.5 × TBE. The products were visualized by a VersaDocTM MP 1000 system (Bio-Rad).

### 3.7. DNA Helicase Assay

Reaction mixtures (10 μL) containing 5 pmol of fluorescent substrate and the indicated amounts of proteins in helicase assay buffer (200 mM HEPES/NaOH, pH 7.5, 10 mM β-mercaptoethanol, 300 mM NaCl, 20 mM ATP, 20 mM MgCl_2_) were incubated for 30 min at 60 °C in a heated-top PCR machine to prevent evaporation. Reactions were stopped by addition of 5 μL of 5 × Stop Solution (0.5% SDS, 40 mM EDTA, 0.5 mg/mL proteinase K, 20% glycerol, 0.1% bromophenol blue) and the products separated on 8% polyacrylamide gel (19:1) in 1 × TBE, containing 0.1% SDS at a constant voltage of 100 V. After electrophoresis, the gels were visualized by a VersaDoc^TM^ MP 4000 system (Bio-Rad) and quantified using ImageJ 1.5 as the processing program. The enzymatic product values were calculated considering the intensity of the product versus the intensity of the remaining substrate.

### 3.8. Absorption Spectroscopy

Absorption spectra in the UV-visible region were performed on a J1100ST spectrophotometer (Jasco, Tokyo, Japan) in quartz cuvettes of different volumes, in 10 mM sodium phosphate buffer pH 8.0.

### 3.9. Labelling of NurA, HerA and SSB with Fluorescein and Rhodamine

A solution of 1 mg/mL NurA in 1 mM sodium phosphate buffer pH 7.0 was reacted with a threefold excess of fluorescein 5(6)-isothiocyanate in DMSO for 1 h at 37 °C. A solution of 0.5 mg/mL HerA and 1 mg/mL SSB in 1 mM sodium phosphate buffer pH 7.0 were reacted with a threefold excess of rhodamine B in DMSO for 1 h at 37 °C. The resulting labeled proteins were separated from the free dye by Sephadex G-25 column. Measurements of absorption between 400 nm and 220 nm of each fraction were achieved to detect the conjugates. The eluted fractions, corresponding to the first fluorescent volume excluded, represented the labeled proteins. The fractions containing the conjugates were pooled, and a final spectrum with absorption of 0.1 O.D. was obtained to determine the degree of labeling and to perform the Forster Resonance Energy Transfer (FRET) analyses.

### 3.10. Determination of the Degree of Labeling

The relative efficiency of the labeling reaction was determined by measuring the absorbance of the protein at 280 nm and the absorbance of the dyes at their absorbance maximum. Using the Beer–Lambert law, the approximate number of dye molecules per protein molecule was calculated. Initially, the protein concentration was determined and corrected for the contribution of the dye to the absorbance at A_280_

Correction factor (CF) = A_280_ free dye/A_max_ free dye (1)

A_protein_ = A_280_ − (A_280_ × CF) (2)

The protein concentration was calculated, and the degree of labeling (DOL) was determined:

DOL = A_max_ × MW/[protein] × E_dye_ (3)

in which A_max_ is the maximum absorbance of dyes, MW is the molecular weight, E_dye_ is the extinction coefficient of the dyes at their absorbance maximum and the protein concentration is expressed as mg/mL.

### 3.11. Fluorescence Spectroscopy

Steady-state fluorescence measurements were performed on an FP8200 spectrofluorometer (Jasco, Tokyo, Japan) equipped with a temperature-controlled sample holder in a quartz cell of 400 µL volume. Fluorescein 5(6)-isothiocyanate was excited at 495 nm with the slit width of 5 nm, and emission spectra were recorded in the range 500–600 nm with the slit width of 5 nm. Fluorescence spectra were measured in 10 mM sodium phosphate buffer pH 8.0. To perform FRET measurements, fluorescein 5(6)-isothiocyanate and rhodamine B were selected as donor-acceptor pair fluorophores because fluorescence emission of the donor overlaps with the absorption/excitation spectrum of the acceptor. The use of FRET between the two fluorophores allows for fluorescence spectral variations since FRET is a through-space interaction that occurs whenever the donor and the acceptor are within the Forster distance (R_0_) and does not require a change in the probe microenvironment.

## 4. Conclusions

In all organisms, genomic DNA is continuously subjected to a wide variety of lesions; among the various types of DNA lesions, double-strand breaks (DSBs) are one of the most harmful. In eukaryotic cells, two major DSB repair pathways are known: Non-Homologous End Joining (NHEJ) and Homologous Recombination (HR). HR is one of the most important DSB repair pathways and, in contrast to NHEJ, it is a high-fidelity mechanism since it relies upon homologous DNA sequences and generates error-free repaired products. In all organisms, initiation of homologous recombination requires the processing of DNA ends in 3′ overhangs, which are necessary for recombinase loading and subsequent strand invasion. This process needs many interactive and regulated proteins and is highly studied in bacteria and eukarya, but is poorly understood in archaea [47].

In archaea, several homologous eukaryotic HR proteins have been identified, including MRE11, RAD50 and the recombinase RadA. Moreover, two archaeal genes, HerA and NurA, usually located in operons, have been implicated in HR because of their conserved genomic association with mre11 and rad50 [20]. It has been suggested that these two proteins may be the functional homologs of the eukaryotic Dna2/DNA2, Exo1/EXO1 and Sgs1/BLM proteins [47]. We have previously shown that *S. solfataricus* NurA is endowed with exonuclease activity and ssDNA nicking activity. Moreover, we demonstrated that NurA interacts with HerA forming a stable complex that shows an even stronger exonuclease activity. Furthermore, we showed that the two activities are located on different catalytic sites of the proteins [28]. In order to get a deeper insight into the DNA end resection mechanism in archaea, we decided to identify possible functional partners of NurA. In 2008, Wei et al. demonstrated that *Sulfolobus tokodaii* StoSSB interacts with StoNurA; moreover, they found that StoSSB inhibited the 5′-3′ ssDNA and dsDNA exonuclease and ssDNA endonuclease activities of StoNurA, suggesting that the two proteins may function closely together in archaeal DNA end resection [39]. Based on this observation and on the reported data on RPA role on Dna2, Sgs1/BLM or WRN activities [7,11,15,48], we decided to analyze the role of SSB protein in *S. solfataricurs* DNA end resection. These studies are particularly interesting since SSB/RPA proteins are present in all domains of life and play critical roles in many genomic processes (such as DNA replication, repair and recombination); furthermore, they bind ssDNA with high affinity and in a sequence-independent manner and, in doing so, helps to form the central nucleoprotein complex substrate for DNA replication, recombination and repair processes. Aside from stabilizing ssDNA structures, as regulatory factors, they also stimulate or inhibit the activities of many proteins involved in DNA metabolism. SSB/RPA can act with two distinct regulation mechanisms: (i) through protein-protein interactions and (ii) through protein-DNA interactions by binding ssDNA and affecting the substrate topology.

**Figure 9:**
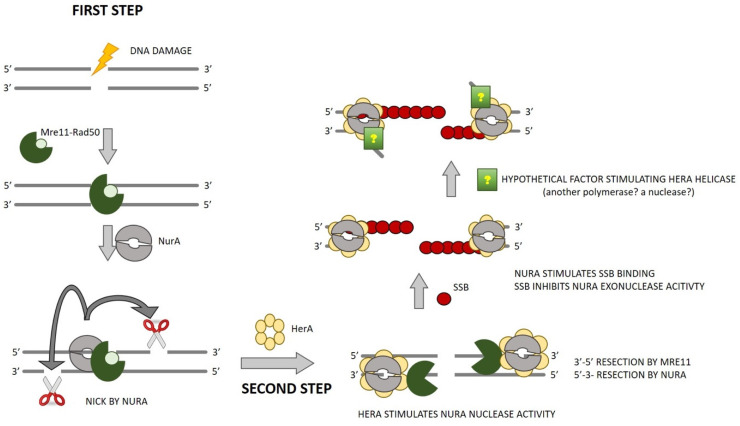
Two-step end resection model in *S. solfataricus*. First step: Mre11/Rad50 (dark and light green, respectively) complex recognizes and binds the DSB. NurA (grey dimer) nicks the DNA strand that possesses a free 5′ end giving rise to a downstream step of resection. Second step: a 3′ ssDNA is generated in a bidirectional manner using the 3′-5′ and 5′-3′ exonuclease activities of MRE11 and NurA, respectively, together with HerA (yellow hexamer) that stimulates NurA exonuclease activity. SSB (red circle) is now rapidly recruited to the DNA, binds to ssDNA generated by the combined action of NurA and Mer11 and forms a nucleoprotein filament that inhibits NurA exonuclease activity. At this step, another protein has to be involved (such as polymerase, nuclease or recombinase, represented as a green square with a yellow question mark) in order to enhance HerA helicase activity and the subsequent recombination process.

Taken together, our results suggest that in archaea, the activities of NurA are selectively modulated by other factors during the DNA end resection process in order to produce appropriate 3′ overhangs for the subsequent loading of other recombination enzymes. We propose that the first event in the DNA end resection process of archaea is Mre11/Rad50 recognition of the DNA damage (Figure 9).

The binding of Mre11/Rad50 onto the damaged DNA causes the recruitment of NurA that nicks the DNA strand that possesses a free 5′ end. The generation of the initial single-strand nick gives rise to the downstream step of resection that starts with the binding of Her to NurA in order to promote its 5′-3′ exonuclease activity. While single-strand DNA is generated by the combined action of Mre11 and NurA, SSB binding to DNA is drastically stimulated and causes the inhibition of NurA exonuclease. Most probably, at this step, another protein has to be involved (such as polymerase, nuclease or recombinase) in order to enhance HerA helicase activity and the subsequent recombination process (Figure 9). Nevertheless, more studies are needed to confirm this hypothetical model and to get more insight into molecular and cellular functions of NurA/HerA in DNA metabolism in archaea.

## Data Availability

The data presented in this study are available on request from the corresponding author.

## Supplementary Materials

The following are available online at https://www.mdpi.com/article/10.3390/ijms23052582/s1.
