# Supplementary material for: The Finely Coordinated Action of SSB and NurA/HerA Complex Strictly Regulates the DNA End Resection Process in Saccharolobus solfataricus
Source: Int J Mol Sci. 2022 Feb 26;23(5):2582. doi: 10.3390/ijms23052582 (PMC8910471; doi:10.3390/ijms23052582)
Supplement: Supplementary file 1 [file ijms-23-02582-s001.zip › ijms-1601803-supplementary.pdf]

The Finely Coordinated Action of SSB and NurA/HerA Complex Strictly Regulates the DNA End Resection Process in *Saccharolobus solfataricus*

Mariarosaria De Falco [1,*], Alessandra Porritiello [1], Federica Rota [1], Viviana Scognamiglio [2], Amina Antonacci [2], Giovanni del Monaco [1] and Mariarita De Felice [1,*]

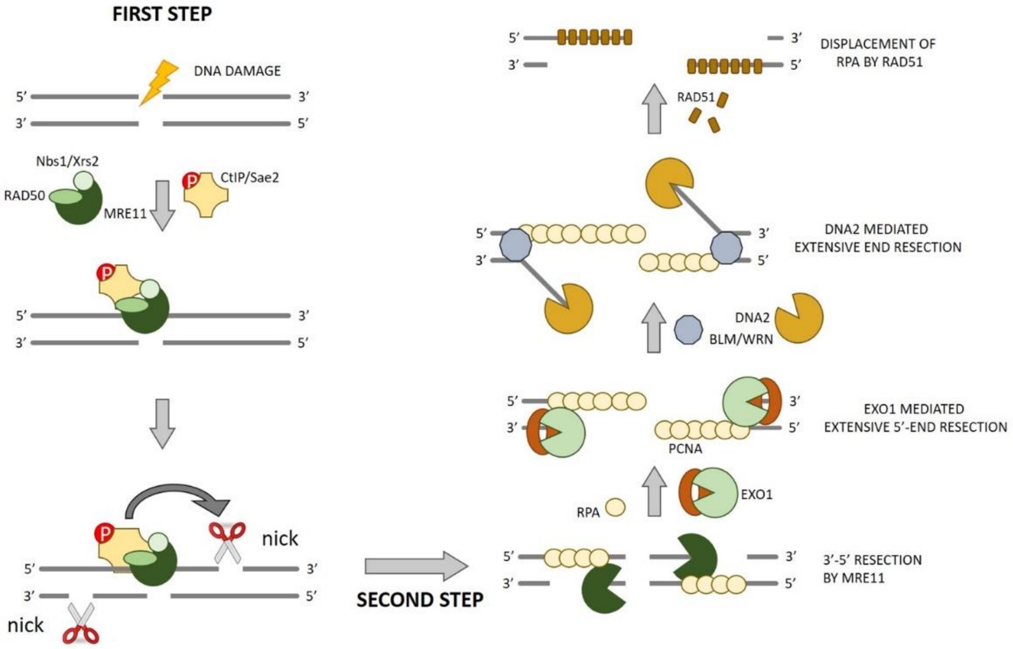

Figure S1. Two steps end resection model. First step: MRN/MRX complex (Mre11/Rad50/Nbs1 or Mre11/Rad50/Xrs2) recognizes and binds the DSB. MRE11, combined with CtIP/Sae2, nicks the DNA strand that possesses a free 5′ end up to 300 nt internal to the DNA, from which starts a downstream step of resection. Second step: a 3′-ssDNA is generated in a bidirectional manner using the 3′-5′ and 5′-3′ exonuclease activities of MRE11 and EXO1, respectively, together with BLM and DNA2. Rad51 binds to the 3′ overhangs, forming a nucleoprotein filament that is responsible for the homology search and invasion step.

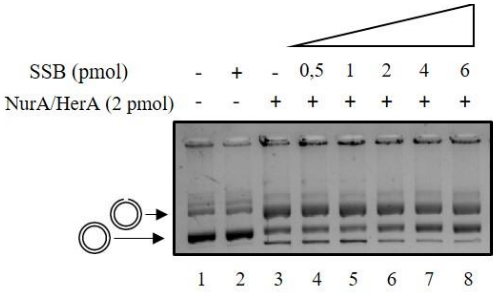

Figure S2. NurA/HerA nicking activity is inhibited by SSB. NurA/HerA nicking activity has been tested on a pcDNA plasmid as circular double stranded DNA substrate. Negative controls were performed in absence of proteins (lane 1) and in the presence of 6 pmol of SSB alone (lane 2). NurA/HerA endonuclease activity was analyzed in the absence (lane 3) and in the presence of increasing amounts of SSB (lanes 4-8).

PROTEIN FOLDING ANALYSIS

Materials and methods

Circular Dichroism Spectroscopy

Circular dichroism (CD) spectra were measured under a nitrogen flow on a J-110 Spectropolarimeter (Jasco, Tokyo, Japan) equipped with temperature-controlled system. Sealed cuvettes with a 1 cm path length (Helma, Jamaica, NJ) were used. During the measurement the photomultiplier voltage never exceeded 600 V. All spectra were recorded in the range of 200 - 240 nm with a resolution of 1 nm, scan rate of 50 nm min^{-1}, band width of 1 nm e and time response of 2 seconds. The parameters were chosen to obtain spectra without any distortion and with the best ration signal-to-noise. The spectra were smoothed with the Spectropolarimeter System Software (Jasco, Japan). CD analysis in far UV region were performed on samples with a concentration of 1 mg/ml for NurA, 0.5 for HerA mg/ml, and 1 mg/ml for SSB while analysis in 1 mM Sodium Phosphate buffer pH 7.0.

Results and discussion

Crystal structures of NurA, HerA, and SSB are well known in the literature with code 2YGK, 4D2I, and 1O7I in Protein Data Bank (PDB), respectively. In particular, these proteins show a typical α/β structure. Such structures were confirmed by circular dichroism (CD) analyses that aided to achieve information about the secondary and the tertiary structure of the proteins. In particular, Figure 9 A reports the crystal structures of NurA, HerA, and SSB and Figure 9 B shows their CD spectra in the far-UV region at 25 °C. In particular, NurA shows a typical mixed α-helix and β-sheet structure with two peaks centered at 212 and 222 nm, as also confirmed by the crystal structure present in PDB, as well as HerA, with two peaks centered at 211 and 222 nm. On the contrary, SSB shows a typical β-sheet structure with a single peak that dominates at 213 nm. The results obtained suggest that the three proteins are in a correct three-dimensional conformation, indicating that the absence of DNA helicase activity cannot be attributed to a mis-folding of HerA and its interacting proteins.

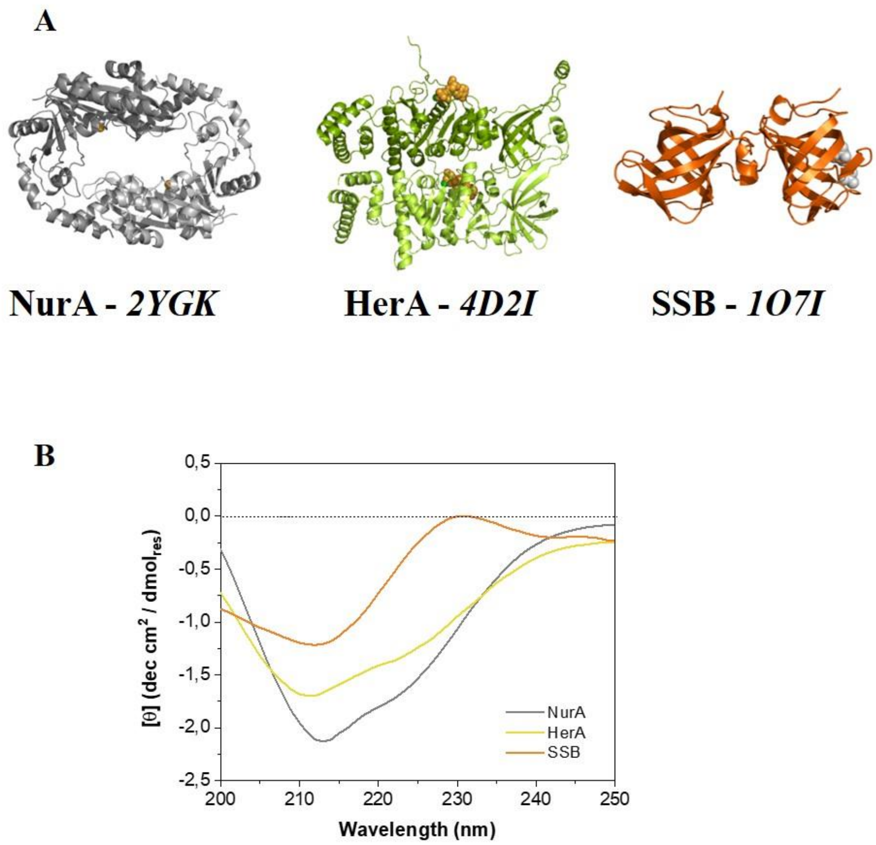

Figure S3. A) Crystal structures of NurA, HerA, and SSB from PDB. B) Circular dichroism spectra of NurA, HerA, and SSB. Buffer: 1 mM Sodium Phosphate buffer pH 7.0.
